# The Mechanism of Energy Coupling in H^+^/Na^+^-Pumping Membrane Pyrophosphatase—Possibilities and Probabilities

**DOI:** 10.3390/ijms23169504

**Published:** 2022-08-22

**Authors:** Alexander A. Baykov, Viktor A. Anashkin, Anssi M. Malinen, Alexander V. Bogachev

**Affiliations:** 1 Belozersky Institute of Physico-Chemical Biology, Lomonosov Moscow State University, Moscow 119899, Russia; 2 Department of Life Technologies, University of Turku, FIN-20014 Turku, Finland

**Keywords:** membrane pyrophosphatase, active transport, H^+^ pump, Na^+^ pump, energy coupling, direct coupling, pyrophosphate, cooperativity

## Abstract

Membrane pyrophosphatases (mPPases) found in plant vacuoles and some prokaryotes and protists are ancient cation pumps that couple pyrophosphate hydrolysis with the H^+^ and/or Na^+^ transport out of the cytoplasm. Because this function is reversible, mPPases play a role in maintaining the level of cytoplasmic pyrophosphate, a known regulator of numerous metabolic reactions. mPPases arouse interest because they are among the simplest membrane transporters and have no homologs among known ion pumps. Detailed phylogenetic studies have revealed various subtypes of mPPases and suggested their roles in the evolution of the “sodium” and “proton” bioenergetics. This treatise focuses on the mechanistic aspects of the transport reaction, namely, the coupling step, the role of the chemically produced proton, subunit cooperation, and the relationship between the proton and sodium ion transport. The available data identify H^+^-PPases as the first non-oxidoreductase pump with a “direct-coupling” mechanism, i.e., the transported proton is produced in the coupled chemical reaction. They also support a “billiard” hypothesis, which unifies the H^+^ and Na^+^ transport mechanisms in mPPase and, probably, other transporters.

## 1. Introduction

Integral membrane pyrophosphatase (mPPase), first described sixty years ago in the bacterium *Rhodospirillum rubrum* [1], is found in all plants and some bacteria, archaea, and protists [2,3,4,5,6]. mPPase is a reversible cation pump that hydrolyzes PP_i_ to build up electrochemical potential gradients of monovalent cations, unlike ubiquitous soluble PPases, which dissipate PP_i_ energy as heat. The presence of mPPase is associated with increased tolerance to abiotic stress, such as drought and salinity [6,7].

For a long time, mPPases were believed to pump only protons and were accordingly referred to as H^+^-PPases. In 2005, the first mPPase with an absolute requirement for Na^+^ (Na^+^-PPase) was discovered in the bacterium *Thermotoga maritima* [8], followed by a demonstration that this and similar Na^+^-dependent mPPases from *Methanosarcina mazei* [9] and other prokaryotes [10,11] pump Na^+^. Later studies have revealed that Na^+^-PPases act in addition as H^+^ pumps, some only at sub-physiological Na^+^ concentrations (<10 mM) [12], and some (named Na^+^,H^+^-PPases) even in the presence of 100 mM Na^+^ [13]. All mPPases absolutely require Mg^2+^ ions for activity but are divided into two homologous families, depending on whether K^+^ acts as an activator or not [14]. K^+^-dependent mPPases contain Ala at the position occupied by Lys in K^+^-independent H^+^-PPases [14]. The positively charged Lys amino group replaces K^+^ in the latter mPPases [15]. K^+^ partially neutralizes the negative charge on substrate oxygen [15], which explains K^+^ effects on *k*_cat_. Additionally, Na^+^ activates Na^+^-transporting mPPases at 1–2 orders of magnitude lower concentrations in the presence of K^+^ [8,9,10,12,13,16,17,18]. All characterized Na^+^-pumping mPPases belong to the K^+^-dependent family, whereas H^+^-pumping PPases are found in both families. All mPPases are formed by two identical subunits (650–980 amino acid residues each), typically folded into 16 or 17 transmembrane α-helices.

**Figure 1:**
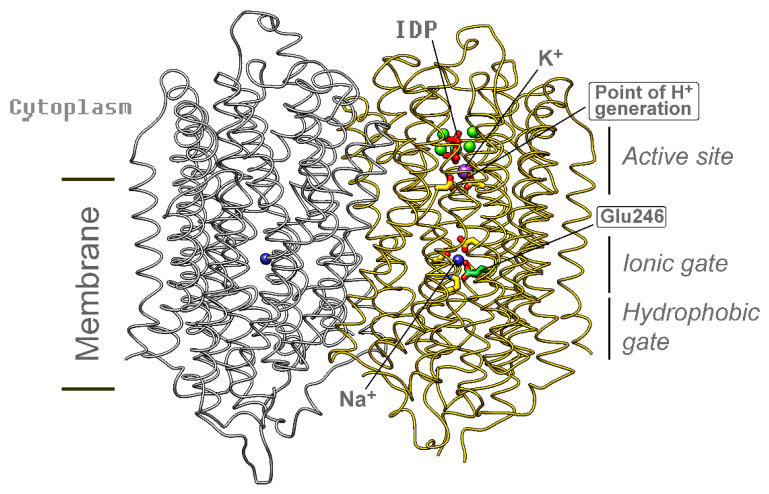
The structure of *T. maritima* Na^+^-PPase homodimer with bound IDP (red) and metal ions (Mg^2+^, green; K^+^, magenta; Na^+^, blue) (PDB code: 6QXA; chain B) [21]. Bound IDP and Mg^2+^ ions are removed from the left subunit. Two nucleophile-coordinating and three ionic gate-forming amino acid residues are shown by sticks. The figure was created with the program UCSF Chimera [22].

In 2012, the 3D structures of the K^+^-dependent H^+^-PPase from the plant *Vigna radiata* (Vr-mPPase) [19] and Na^+^-PPase of *T. maritima* (Tm-mPPase) [20] were reported. These structures indicated that both transporters form dimers of identical subunits, each having its own catalytic and transport machinery (Figure 1). The catalytic site is a large cavity on the cytoplasmic side of the membrane, accommodating five Mg^2+^ ions, one K^+^ ion, and a molecule of imidodiphosphate (IDP), a remarkably close non-hydrolyzable PP_i_ analog with an NH group replacing the bridge oxygen atom [15,19]. Tm-mPPase structure contains in addition one Na^+^ ion [15]. The active site is connected to the vacuolar lumen/periplasmic space via an ion-conducting channel formed by six internal α-helices. The active site and channel structures are very similar in the two pumps despite their different ion specificities, except for the relocation of a conserved gate Glu residue one helix turn closer to the active site in Na^+^-PPase. This residue is Glu246 in Tm-mPPase (E^6.53^ in the Ballesteros–Weinstein residue notation [3]). Sequence comparisons confirmed that different positioning of the Glu residue delineates Na^+^-PPases from K^+^-dependent H^+^-PPases. However, K^+^-independent mPPases operate as H^+^ pumps even though the Glu is positioned in them like in Na^+^-PPases.

mPPases have no homologs among known ion pumps, implying a unique coupling mechanism that unifies H^+^ and Na^+^ transport. However, the existing views on this mechanism are highly controversial. Areas where substantial differences have been observed include the identity of the coupling step, the fate of the proton produced in PP_i_ hydrolysis, the role of the quaternary structure, and the relation between H^+^ and Na^+^ transport. Work in the past decade provided important data of a mechanistic nature to support the “billiard” hypothesis of mPPase functioning put forward in a review publication of 2013 [2]. This hypothesis centers on the proton produced from the water nucleophilic as the key element of the mPPase transport mechanism. Here, we critically review the recent data, including those related to the “billiard”-type mechanism of mPPase, and pave the way for its future studies.

## 2. The Coupling Step

**Scheme 1:**
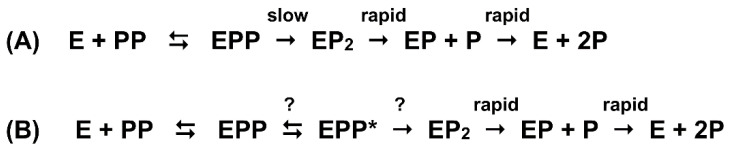
Kinetic models of PP_i_ hydrolysis by mPPase. (**A**) A minimal four-step model. (**B**) A five-step model incorporating a conformational change on the main route. H^+^ transport occurs concurrently with the EPP → EPP* or EPP* → EP_2_ conversion.

PP_i_ hydrolysis by mPPase involves at least four steps: PP_i_ binding, bound PP_i_ hydrolysis, and stepwise release of two phosphate (P_i_) molecules (Scheme 1A). Of note, it is commonly accepted that the actual substrate of mPPases is the Mg_2_PP_i_ complex, referred to from here on briefly as PP_i_. Accordingly, the two P_i_s leave the active site as mono-magnesium complexes. Recent pulse-chase measurements of PP_i_ binding to Tm-mPPase have revealed that the reaction sequence additionally involves enzyme isomerization before the hydrolysis step [23], as in soluble pyrophosphatase [24]. A corresponding extended variant of the reaction sequence is shown in Scheme 1B. In principle, any of the first two steps in model A or three steps in model B might provide energy for cation transport, i.e., be the coupling step. A common strategy used to identify coupling steps in membrane pumps consists of comparing the kinetics of the chemical and transport reactions. Ideally, the chemical reaction is dissected into individual steps, and their rates are estimated and compared with the rate of solute transfer across the membrane. This approach has been particularly useful with the transporters coupled to redox reactions whose kinetics can be monitored by visible light spectroscopy. mPPase reaction does not involve usable spectral changes, hampering the evaluation of the rates of its individual steps.

**Figure 2:**
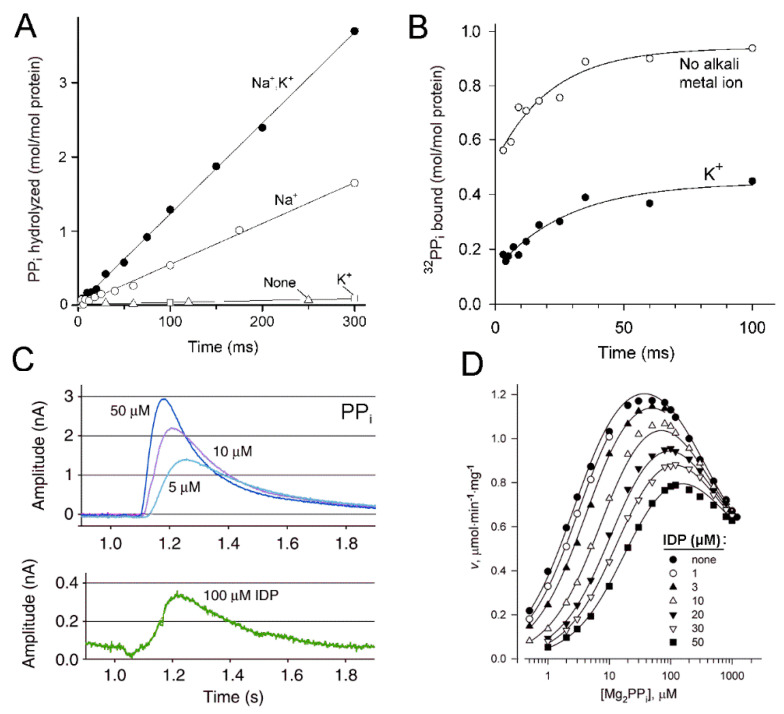
Selected mechanism-deciphering functional data. (**A**) Pre-steady-state kinetics of PP_i_ hydrolysis by Tm-mPPase (4.3 µM enzyme, 79 µM ^32^PP_i_). The curve labels refer to the alkali cations present (none, K^+^ only, Na^+^ only, or both Na^+^ and K^+^). (**B**) Pulse-chase measurements of the rapid kinetics of ^32^PP_i_ binding to Tm-mPPase in the absence of Na^+^ (0.8 µM enzyme, 2.5 µM ^32^PP_i_). The binding reaction was arrested after the indicated time by adding excess nonlabelled PP_i_ and NaCl, the bound ^32^PP_i_ was hydrolyzed for 1 s, and the amount of ^32^P_i_ in the system was measured after acid quench. The alkali metal ion present during the binding step is indicated on the curves. Panels A and B were reproduced from Malinen et al. [23] under the terms of the Creative Commons CC BY license. (**C**) Electrometric traces of *V. radiata* pyrophosphatase-loaded liposomes obtained using a Nanion SURFE^2^R N1 instrument. Currents were recorded following the addition of 5, 10, or 50 µM K_4_PP_i_ (upper part) or 100 µM IDP (lower part). Data were reproduced from Li et al. [15] under the terms of the Creative Commons CC BY license. (**D**) Steady-state kinetics of PP_i_ hydrolysis by Dh-mPPase in the absence and presence of the indicated concentrations of IDP. The panel was reproduced from Anashkin et al. [25] under the terms of the Creative Commons CC BY license.

Rapid kinetics of PP_i_ hydrolysis was measured only for *T. maritima* Na^+^-PPase [23]. Quenched-flow measurements of P_i_ formation during the first 300 ms of the hydrolysis reaction, corresponding to approximately three turnovers, after a rapid (<5 ms) mixing of mPPase with a large excess of PP_i_ in the presence of Na^+^ or Na^+^ and K^+^ yielded linear product versus time dependencies [23] (Figure 2A). No PP_i_ hydrolysis was observed in the absence of Na^+^, irrespective of whether K^+^ was present or absent. Because the product assay method used measured the sum of the medium and enzyme-bound P_i_, the simplest explanation of the linear product formation in terms of Scheme 1A is that the enzymatic reaction is limited by the Na^+^-dependent step of bound PP_i_ hydrolysis. A product burst would indicate a significant contribution of the product release steps, whereas a lag would indicate the contribution of a preceding conformational change. If the reaction involves a conformational change in the enzyme–substrate complex (Scheme 1B) [23], the hydrolysis step may be similarly rate-limiting and all other steps, including the conformational change, very fast. Alternatively, the EPP → EPP* conversion may be rate-limiting and all subsequent steps, including bound PP_i_ breakdown, be much faster. Both alternatives are consistent with the linear pre-steady-state kinetics, but the mechanism with the rate-limiting chemical step is also favored by the high kinetic D/H isotope effect determined by comparing hydrolysis rates in D_2_O and H_2_O [23].

Energy coupling most likely occurs at the rate-limiting step. Hence, the transported cation (Na^+^ in Tm-mPPase) should bind before or during the rate-limiting step but not after it. Pulse-chase measurements indicated no Na^+^ requirement for PP_i_ binding by Tm-mPPase [23]. In these experiments, the enzyme was rapidly mixed with a small amount of ^32^PP_i_ in the absence of Na^+^ to disallow PP_i_ hydrolysis, and the ^32^PP_i_ binding reaction was allowed to proceed for 5–100 ms before it was arrested by adding an excess of nonlabelled PP_i_ (450 µM final concentration) and NaCl. After 1 s, the time sufficient to hydrolyze all bound ^32^PP_i_, the mixture was quenched with acid, and the amount of the initially bound ^32^PP_i_ was estimated from the amount of ^32^P_i_ formed. Rapid binding of up to 0.75 mol/mol ^32^PP_i_ was observed in these experiments in the absence of Na^+^, the transported ion (Figure 2B), consistent with the results of the steady-state kinetic analysis with *M. mazei* Na^+^-PPase (Mm-mPPase) [18]. These findings ruled out PP_i_ binding as the coupling step for both models in Scheme 1.

The pulse-chase measurements additionally indicated that PP_i_ binding to Tm-mPPase in the absence of Na^+^ occurs as a rapid, diffusion-controlled process followed by a slow isomerization step (conformational change) [23]. The rate constant for the latter step exceeded several-fold the catalytic constant measured in the presence of Na^+^. However, the isomerization rate constant should also be measured in the presence of Na^+^ for a direct comparison with the catalytic constant.

Rapid kinetics of charge transfer was measured for a different transporter—H^+^-pumping Vr-mPPase. Li et al. [15] and Shah et al. [26] used a Nanion SURFE^2^R instrument to assay transmembrane H^+^ transfer by Vr-mPPase. mPPase-harboring liposomes were adsorbed to a solid membrane on a gold sensor chip, and the electric current was measured upon rapid supply of the substrate PP_i_ via a microfluidic system. The claimed dead-time of the instrument is 10 ms, which is 1/10th of the time required for one enzyme turnover because mPPase is a relatively slow enzyme (*k*_cat_ for Vr-mPPase is 12 s^−1^ [5]). This experiment aimed at measuring the currents generated by binding of three non-hydrolyzable PP_i_ analogs and testing in this way the possibility that H^+^ transport is coupled to substrate (Mg_2_PP_i_ complex) binding. The analogs included IDP and two diphosphonates, having CH_2_ or CH(OH)CH_3_ groups in the bridge position instead of the oxygen atom. The small currents observed with IDP and one diphosphonate analog led the authors to claim that H^+^ transport occurs at the substrate binding step [15,26]. However, this interpretation is refuted by the observed 10 times greater electrometric signal generated by PP_i_ within the time sufficient for only one mPPase turnover [15,26]. Together, these data unambiguously indicate that proton transport occurs in a step following substrate binding in the reaction sequence [27].

This conclusion is supported by the finding that the peak current, which is proportional to the rate of proton transfer, reached a constant value with increasing PP_i_ concentration, with a half-maximal effect at approximately 5 µM [15], a value similar to the Michaelis constant for PP_i_ hydrolysis (3–5 µM [16]). Furthermore, the possibility that the signal results from PP_i_ binding is inconsistent with only a small dependence of the position of the signal maximum on the time axis on PP_i_ concentration [15]. PP_i_ binding is a second-order reaction, and its rate must increase linearly with PP_i_ concentration. Larger gate-pore size in the 2P_i_-bound state (3.7 Å) than in the IDP- and P_i_-bound states (2.0–2.1 Å) in the crystal structures [28] is also consistent with H^+^ transport immediately following PP_i_ hydrolysis.

## 3. Subunit Cooperation

Recent studies have demonstrated significant functional and structural differences in two chemically identical subunits of mPPase, which may relate to the energy-coupling mechanism. Although each subunit has its own active site, binding of the second substrate molecule decreases rather than doubles the hydrolysis rate [17,21,25]. Moreover, the Michaelis constant for interaction with the second substrate molecule is greater by two orders of magnitude [17,25]. Consequently, at any given time, only one subunit of dimeric mPPase predominantly binds PP_i_ at its concentrations less than 100 µM. Structural asymmetry of mPPase was demonstrated by X-ray crystallography of its complex with an isoxazole derivative (allosteric inhibitor)—its two copies bound to only one subunit [21]. Molecular dynamics simulations of Vr-mPPase–IDP complexes of different stoichiometries indicated that IDP binding to one subunit induces larger conformational changes in the other subunit, and these changes are not compensated in bi-IDP complex [25].

**Figure 3:**
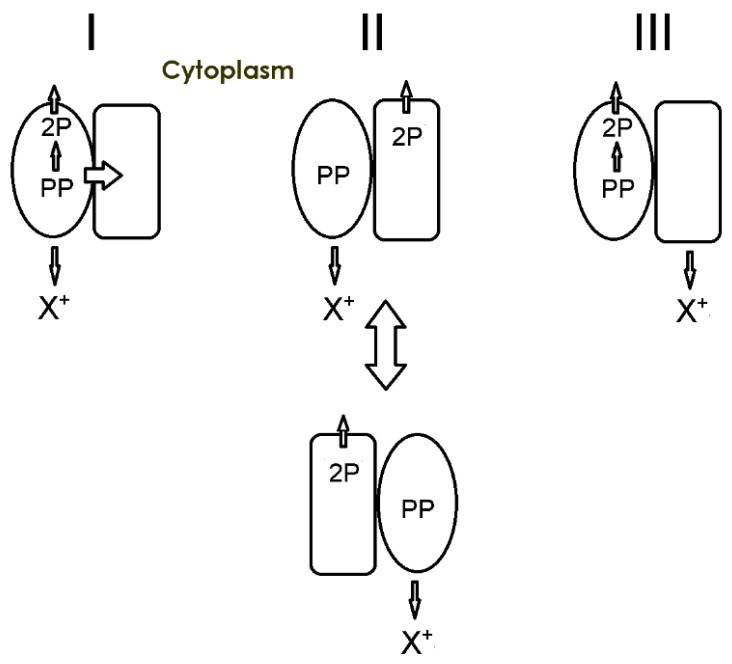
Three possible ways (I–III) of subunit cooperation in cation transport by mPPase. X is the transported H^+^ or Na^+^ ion.

This asymmetry may have several mechanistic explanations (Figure 3). In Mechanism I, both hydrolysis and transport events occur in the same subunit, and the other subunit plays an auxiliary role, e.g., in transient energy storage [29]; subunit roles may interchange in different catalytic cycles. This simplest mechanism is consistent with the currently available data and will remain the main working hypothesis until disproved by new data. The other possibility is that hydrolysis and transport proceed in one subunit, but product release requires PP_i_ binding to the other subunit (Mechanism II). Consequently, subunits work alternately in this mechanism. Still another possibility is that hydrolysis and transport are carried out in different subunits in each reaction cycle (Mechanism III). In this mechanism, the transport of the gate-bound ion (H^+^ or Na^+^) in the substrate-free subunit is triggered by a conformational change induced by PP_i_ hydrolysis or a conformational change in the other subunit. Na^+^ may reach the gate site from the cytoplasm more easily in Mechanism III because the transporting subunit never contains PP_i_, which may interfere with Na^+^ binding.

An alternative scheme of subunit cooperation in transport and hydrolysis was proposed by Goldman’s group [21,30]. This scheme assumed that substrate binding to one subunit potentiated its binding to the other and was based on the observation of a sigmoidal activity versus substrate concentration profile for Tm-mPPase [21]. We never observed sigmoidicity with a dozen mPPases, including Tm-mPPase [23], and believe that our kinetic data are more accurate at low substrate concentrations. The enzyme assay we used is continuous and, hence, more sensitive [31], a critical point in the kinetic analysis of low-*K*_m_ enzymes [32].

## 4. Direct or Indirect Coupling?

In the “direct-coupling” mechanism, the translocated charge is an integrant part of the substrate or product. The charges involved in all so far characterized “directly coupled” pumps are either electrons or protons, and the chemical reaction is always an oxidation/reduction [33]. Nearly 50 years ago, Peter Mitchell [34] proposed a “direct-coupling” mechanism for a non-oxidoreductase transporter F_o_F_1_-ATP synthase, which is functionally similar to mPPase. However, this mechanism was confuted by higher than 1:1 H^+^/ATP transport stoichiometry demonstrated for this enzyme. Furthermore, 3D structural analysis of F_o_F_1_ ATP synthase placed the catalytic site and the translocation channel far apart [35], confirming that they interact with each other indirectly via a long-range conformational change [36]. Accordingly, the transported proton comes from the medium, whereas the “chemical” proton is dissipated in the medium.

In mPPase, the active site and ion-conducting channel are in close proximity [19,20]. The proton is generated from the nucleophilic water, the second substrate, just at the channel entrance, which led Lin et al. [19] to propose a “direct-coupling” mechanism for this pump, in which the “chemical” proton enters the ion-conducting channel and moves through it via a proton “wire” formed by *n* water molecules. Although a proton appears on the other membrane side at each catalytic cycle, *n* + 1 cycles are required for a particular proton to pass through the ion conductance channel. In contrast, Na^+^ is not a reaction product and should be occluded by mPPase before the coupling step. PP_i_ binding causes active site closure by the loop connecting α-helices 5 and 6 [15,20]; hence, Na^+^ may be occluded during the PP_i_ binding/conformational change steps in Scheme 1B. The structure in Figure 1 refers thus to the occluded state. Consistent with the “direct-coupling” mechanism, most studies indicated a required 1:1 H^+^/PP_i_ stoichiometry for H^+^-PPase [37,38,39].

PP_i_ hydrolysis in the presence of Mg^2+^ produces practically no protons because the substrate PP_i_ mainly exists as a MgP_2_O_7_^2−^ complex and the product (two P_i_ molecules) as HPO_4_^2−^ and MgHPO_4_ under conditions prevailing in the cytosol [31]. If the “chemical” proton is transported across the membrane, one proton should be captured from the cytoplasm to protonate the product, formally corresponding to a proton transfer from the cytosol across the coupling membrane.

Mechanisms I and II of Figure 3 extend Lin et al.’s formulation to cooperating subunits by assuming that hydrolysis and transport occur in the same way in only one subunit at every moment of time, while the other subunit plays an auxiliary role. The water-generated proton enters the ion-conducting channel causing proton release at the channel exit if the transport is coupled with hydrolysis or reprotonates the gate Glu, which lost its proton during the preceding conformation change in case it is the coupling step. In mechanism III, the coupling is formerly indirect, and the “chemical” proton may dissipate in the cytoplasm or be used to protonate the leaving group phosphate. However, the “chemical” proton may also bind to the gate Glu or another residue in one catalytic cycle and be transported in another cycle in which the subunit roles interchange. In a canonical “indirect-coupling” mechanism (like that of F- or V-type ATPase), the “chemical” proton is never transported.

## 5. The Relationship between H^+^ and Na^+^ Transport

Typical Na^+^-PPases transport both H^+^ and Na^+^ at <10 mM Na^+^ concentration, but only Na^+^ at its higher values [12]. No PP_i_ hydrolysis and, hence, H^+^ and Na^+^ transport occurs without Na^+^ [8,9,12,15,17]. This transport promiscuity raises several questions: (a) Do H^+^ and Na^+^ use the same transport machinery? (b) How is transport specificity regulated by Na^+^ concentration? (c) Why do all Na^+^-transporting mPPases belong to the K^+^-dependent family? The available data provide partial answers to some of these questions.

The structures of Na^+^-PPase [21] (Figure 1) and K^+^-dependent H^+^-PPase [19] are remarkably similar, the most evident difference being in the position of a single Glu residue in the ion-conducting channel, as mentioned above. Both Glu positions allow H^+^ transport, whereas Na^+^ transport requires the Glu residue to be one helix turn closer to the active site to allow the formation of a Na^+^-binding site [21]. The corresponding Glu is also relocated to helix 5 in another mPPase lineage that evolved from Na^+^ to H^+^ pumping [10]. Evidently, there are also yet unrecognized differences, as Glu repositioning alone did not confer Na^+^-transporting activity to H^+^-PPase [15]. Furthermore, K^+^-independent mPPases pump H^+^ despite the fact that the Glu is in the Na^+^-PPase position in the amino acid sequence. However, the pathways of H^+^ and Na^+^ are clearly the same in both mPPase types, consistent with the ability of Na^+^-PPase to transport both cations. Tentative transport mechanisms should also consider that only one subunit binds and converts PP_i_ at any given time in both mPPase types, as discussed above.

The dependence of PP_i_ hydrolysis on Na^+^ concentration for tens of Na^+^-transporting mPPases indicated the involvement of one or two activating Na^+^ ions in the absence of K^+^ and one activating and one inhibitory Na^+^-binding sites in its presence [9,10,12,13,40]. The hydrolysis kinetics of Tm-mPPase demonstrated the involvement of two activating Na^+^ ions in the presence of K^+^, but this abnormal behavior appears to result from a specific difference in the amino acid sequence. Tm-mPPase contains an Aspu residue in the position occupied by Asn in other mPPases; a respective Asp703Asn substitution decreased the number of activating Na^+^ ions to one, irrespective of K^+^ concentration [8]. The available data indicate thus that minimally two Na^+^-binding sites control PP_i_ hydrolysis by Na^+^-transporting mPPases.

The identity of the inhibiting site can be inferred by comparison with H^+^-transporting mPPases. Canonical K^+^- independent H^+^-PPases (having Lys in the K^+^ dependence signature motif) are insensitive to Na^+^ [17], whereas, in canonical K^+^-dependent H^+^-PPases, Na^+^ can act as a less efficient K^+^ substituent [16,41,42,43] and, hence, partially inhibited in its presence. It is thus highly likely that the Na^+^ site manifested in Na^+^-PPases as activating in the absence of K^+^ and inhibiting in its presence [8,10,12,13,17,40] is the K^+^-binding site common to all K^+^-dependent mPPases. This identification is supported by the data showing that Ala → Lys substitutions in the K^+^ dependence signature motif of Na^+^-PPase and Na^+^,H^+^-PPase eliminated their activation by K^+^, without affecting transport specificity [17]. These substitutions thus yielded K^+^-independent Na^+^-PPases, not occurring naturally. It is thus reasonably clear that the bound K^+^ ion and its replacing lysine amino group in K^+^-independent mPPases (the cationic center) do not define the transport specificity of mPPases. Furthermore, the above substitutions eliminated the apparent major effect of K^+^ in Na^+^-transporting mPPases—a profound increase in the binding affinity for the activating Na^+^ ion.

The identity of the “true” activating Na^+^ site with a sub-millimolar binding constant manifested in Na^+^-PPases in the presence of K^+^ is less evident. The crystal structure of Tm-mPPase revealed one gate Glu-bound Na^+^, the putative transported ion [15,21]. This same Na^+^ ion likely cancels H^+^ transport activity at high Na^+^ concentrations by physically blocking “chemical” proton passage through the ion-conducting channel. If so, the gate-Glu site would bear no Na^+^ ion in the H^+^ transport mode and, hence, a different site is required to activate PP_i_ hydrolysis and, consequently, H^+^ transport. The unrecognized activating site may belong to the same subunit or, alternatively, be the gate site of the partner subunit. The latter explanation is consistent with the known subunit interdependence—Na^+^ binding to one subunit may allosterically activate PP_i_ hydrolysis and H^+^ or Na^+^ transport in the Na^+^-free subunit.

Irrespective of the identity of the activating and inhibiting Na^+^ sites, all three mechanisms in Figure 3 could unify Na^+^ and H^+^ transport. Mechanisms I and II accommodate the earlier proposed “billiard-type” mechanism [2,27], which assumes that the “chemical” proton electrostatically displaces the gate-bound Na^+^ ion into the exit channel. In the reverse reaction of PP_i_ synthesis, which might be the major function of mPPase in some organisms [44,45], the back-transported H^+^ will protonate the leaving OH group of the electrophilic phosphate. If the coupling ion is Na^+^, a reverse “billiard-type” mechanism is feasible, in which incoming Na^+^ displaces H^+^ from the channel into the active site. In the less likely Mechanism III, the chemical proton plays no other role than the protonation of the leaving group phosphate in the catalytic subunit while cations are transported in the PP_i_-free subunit. The transport-driving force in Mechanism III could be the conformational energy accumulated in the transporting subunit upon PP_i_ hydrolysis in the catalytic subunit.

PropKa, version 3.4 [46] predicts the p*K*_a_ of the gate Glu246 in Tm-mPPase to be 7.0–7.2 (two values refer to different subunits) in the resting state (PDB structure 4AV3), 8.4–8.5 in the substrate-bound state (PDB structure 5LZQ, Na^+^-depleted), and 10.0 in the “just-after-hydrolysis” state (PDB structure 4AV6 containing two P_i_ molecules per subunit). The difference in the p*K*_a_ values mainly results from changed interactions with two hydrogen-bonding partners—Lys707 and Ser184. Based on the p*K*_a_ estimates, the Glu carboxylate is partially protonated at the neutral pH in the resting state, becomes almost fully protonated after binding the substrate, and keeps the proton in the product-bound state. The Glu protonation driven by the energy released upon substrate binding apparently prepares the proton “wire” for the transport of the “chemical” proton upon PP_i_ hydrolysis.

**Figure 4:**
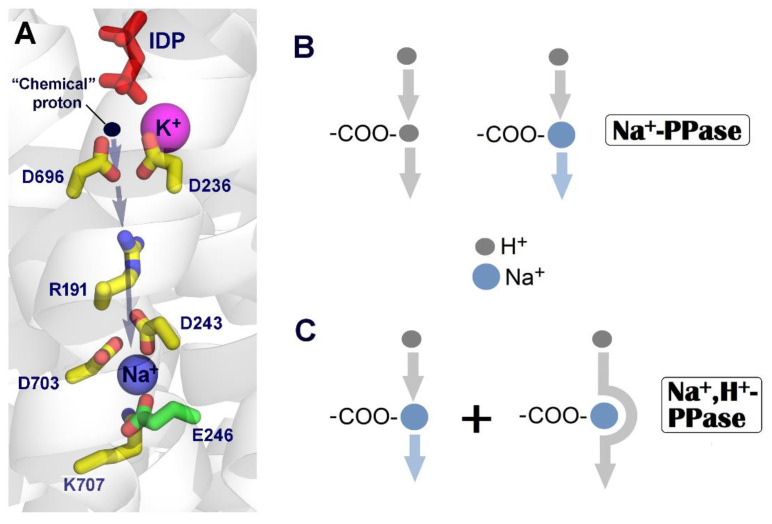
The “billiard-type” mechanism of Na^+^ transport by mPPases. (**A**) Part of the ion conductance channel between the active site and ionic gate in *T. maritima* Na^+^-PPase with bound IDP (red), Na^+^ (blue), and K^+^ (magenta) (PDB code: 5LZQ) [21]. Two nucleophile-coordinating and five channel-forming amino acid residues are shown. The position of the “chemical” proton released from the nucleophilic water molecule was modeled from the structure 4A01. The arrows indicate the presumed path of the “chemical” proton in Na^+^-PPase through the proton “wire” involving in addition several water molecules [19]. The figure was created with PyMOL (The PyMOL Molecular Graphics System, version 1.5.0.4, Schrödinger, LLC, New York, NY, USA). (**B**) “Chemical” proton interactions with the gate-bound H^+^ ion at low Na^+^ a concentration (left) or Na^+^ ion at a high Na^+^ concentration (right) in conventional Na^+^-PPase. The transitory gate site is indicated only by Glu246 carboxylate for simplicity. Proton and Na^+^ motions are shown by gray and blue arrows, respectively. (**C**) Two presumed pathways for the “chemical” proton in Na^+^,H^+^-PPase at a high Na^+^ concentration.

In the Na^+^-transporting mode, the “chemical” proton electrostatically displaces the Na^+^ ion bound to the Glu residue instead of proton into the exit channel (Figure 4A). The identity of the transported cation is thus determined by relative affinities of the gate Glu to Na^+^ and H^+^ (Figure 4B). Noteworthy, PP_i_ hydrolysis does not change the total number of protons in the system because proton release from nucleophilic water is compensated by proton consumption by leaving group phosphate. Accordingly, Na^+^-PPase builds up both membrane electric potential difference (Δψ) and ΔpH in the H^+^ transport mode (at low Na^+^ concentration) [9], whereas only Δψ formation is expected in the Na^+^-only transport mode (at high Na^+^ concentration).

Vr-mPPase transports only H^+^, and its gate Glu301 is located one helix turn further from the active site. The p*K*_a_ value of the Glu carboxylate in the available crystal structures, as predicted by PropKa, is 8.7–8.9 in the substrate-bound and 2P_i_-bound states (PDB structures 4A01 and 6AFS, respectively), consistent with the above estimates for Tm-mPPase. Furthermore, the p*K*_a_ value decreases to 6.4–7.3 in the complex with only the leaving group P_i_ molecule (PDB structure 5GPJ). The latter value is close to that in resting Tm-mPPase, suggesting that it is the electrophilic phosphate residue that increases the p*K*_a_ of the gate Glu residue in mPPase.

## 6. Na^+^,H^+^-Pyrophosphatase and Transport Stoichiometry

It was tacitly assumed in the above treatment that only one cation, H^+^ or Na^+^, is transported per PP_i_ molecule hydrolyzed. This stoichiometry is suggested by the chemical reaction producing only one proton, and the transport machinery is poised to “direct coupling”. Furthermore, this stoichiometry was experimentally demonstrated for a vacuolar H^+^-mPPase [38,39]. In common Na^+^-PPases, H^+^ and Na^+^ are transported in a competitive mode, consistent with this minimal stoichiometry. However, a particular group of bacterial Na^+^-transporting mPPases retain the H^+^ transport function at sub-physiological Na^+^ concentrations, up to 100 mM [13,40]. The lack of competition between H^+^ and Na^+^ suggests that they are both transported in the same reaction cycle. Concurrent Na^+^ and H^+^ transport in Na^+^,H^+^-PPases is, in principle, possible in the frameworks of Mechanisms I and II but may imply a transporΔt stoichiometry (H^+^+Na^+^)/PP_i_ greater than 1. Noteworthy in this regard, Sosa and Celis indeed reported on the H^+^/PP_i_ stoichiometry of 2 in *Rhodospirillum rubrum* H^+^-mPPase [47].

How does such stoichiometry agree with the thermodynamics of the PPase reaction? The number of single-charged cations translocated per 1 PP_i_ molecule hydrolyzed cannot exceed Δ*G*_PP_/Δ$\bar{\mu }$_H_+, where Δ*G*_PP_ is the free energy of PP_i_ hydrolysis and Δ$\bar{\mu }$_H_+ is the proton electrochemical potential difference, given by F × Δ*p*, where F is the Faraday number and Δ*p* is the protonmotive force. The Δ*p* value for cytoplasmic bacterial membrane varies from 140 to 180 mV, depending on growth conditions [48,49,50], corresponding to the Δ$\bar{\mu }$_H_+ range of –13.5 to –17.4 kJ mol^−1^. The total PP_i_ level in bacteria is approximately 1 mM [51], corresponding to Δ*G*_PP_ of –36.7 kJ mol^−1^ provided that all PP_i_ is in the free state. The free PP_i_ concentration in mPPase-containing cells is unknown but evidently represents only a fraction of the total concentration. For instance, in hepatocytes, free PP_i_ (0.4 µM) accounts for only 1/12th of the total PP_i_ [52,53]. Assuming the same PP_i_ distribution in bacteria yields Δ*G*_PP_ = –30.4 kJ mol^−1^, which is still approximately twice Δ$\bar{\mu }$_H_+, permitting thus transfer of two single-charged cations per PP_i_ molecule hydrolyzed under certain conditions. Further work is, however, needed to determine in a more rigorous way Na^+^ and H^+^ gradients and free PP_i_ concentration in bacteria possessing Na^+^,H^+^-mPPase and evaluate the physiological transport stoichiometry of this unique cation pump.

Several mechanistic possibilities to achieve concurrent transport of two monovalent cations in one turnover are envisaged, only some implying the 2:1 transport stoichiometry. One possibility is that the gate site-bound Na^+^ ion only partially blocks “chemical” proton passage to the exit channel in Na^+^,H^+^-PPase, leaving some probability of proton transportation instead of Na^+^, the pathway that is forbidden in Na^+^-PPase (Figure 4C). In billiards terms, “the cue ball ricochets into the hole in some hits.” This mechanism permits the 1:1 transport stoichiometry. The second possibility is that the “chemical” proton follows the displaced Na^+^ ion [13]—“both the hit and the cue balls fall into the hole”. The possibility of Na^+^ and H^+^ transportation by different subunits implies involved coordination between the two catalytic sites to control their cation preferences and seems less likely.

## 7. Comparison with Other Na^+^ Pumps

The available data indicate that the mechanisms of H^+^ and Na^+^ transport by mPPase are unique and different from those used in other ion pumps, including the pumps energized by ATP and other polyphosphates. First, mPPase is the only known H^+^ pump that transports the H^+^ ion formed in the supporting chemical reaction. All “directly coupled” transporters use an electron as the coupling particle [33], and all known H^+^ pumps, including F_O_F_1_-type ATP synthases, Ca^+^-ATPase, and the plasma membrane H^+^-ATPase [54,55,56,57], are based on “indirect-coupling” mechanisms.

Besides being the first non-oxidoreductase “directly coupled” H^+^ pump, mPPase provides a unique solution for associated Na^+^ pumping. Such transport activity is found among several other energy-converting membrane enzymes and proteins, including but not limited to decarboxylases, ATPases, NADH:quinone and ferredoxin:NAD^+^ oxidoreductases, methyltransferases, and rhodopsin [33,58]. Two of them, rotary Na^+^-ATP synthase и Na^+^-rhodopsin, resemble Na^+^-PPase in exhibiting dual Na^+^ and H^+^ transport specificity under certain conditions but exhibit significant differences. The A_O_A_1_ ATP synthase of the archaeon *Methanosarcina acetivorans* similarly catalyzes the concurrent transfer of Na^+^ and H^+^ under physiological conditions [59] but uses an “indirect-coupling” mechanism. More importantly, the transporting membrane-spanning *c* subunit of the ATP synthase is present in multiple copies, whose primary structures may differ [60]. Consequently, Na^+^ transport and H^+^ transport may be associated with different *c* subunits, keeping in mind the variations in the hydrophobicity/polarity balance inside the ion-conducting channel may change its transport selectivity [61]. In Na^+^,H^+^-PPase, produced as a single polypeptide, subunits are identical, necessitating a different explanation of the concurrent transport of the two cations. Furthermore, the Na^+^ and H^+^ transport activities of *M. acetivorans* ATP synthase are not tightly coupled, allowing H^+^ transport and ATP hydrolysis to proceed in the absence of Na^+^ at pH 5 [59]. In contrast, Na^+^,H^+^-PPase retains its Na^+^ transport activity and Na^+^ dependence of hydrolytic activity in acidic media [13].

Bacterial Na^+^-rhodopsin uses a different source of energy but nevertheless resembles Na^+^-PPase more closely because Na^+^ transport is mediated by H^+^ motions in both pumps. In rhodopsins, the H^+^ ion is generated inside the protein molecule by the Schiff base when its p*K*_a_ decreases abruptly due to light-induced retinal isomerization (H^+^-translocating rhodopsin can be therefore considered, with some reservation, a “directly coupled” pump). The cyclical movement of this proton to a nearby Asp residue and back during Na^+^-rhodopsin photocycle allows Na^+^ capture from the cytoplasmic side and its release by a “billiard-type” mechanism on the periplasmic side [62,63,64]. Similar “billiard-type” Na^+^/H^+^ relationships may occur in channel proteins [65].

In contrast, the use of conformational energy is common among membrane pumps. All versions of the mPPase mechanism shown in Figure 3 can be considered evolutionary precursors of the rotary mechanism used in F-, A-, and V-type ATP synthases to store and consume conformational energy. Because PP_i_ hydrolysis involves a smaller free energy change than ATP hydrolysis, mPPase accomplishes this task by means of only two subunits, which oscillate between two conformations, whereas rotation requires at least three subunits. This inference is consistent with the view [66] that PP_i_ preceded ATP as the main energy currency and Na^+^ preceded H^+^ as the coupling ion in the earlier forms of life.

## 8. Perspectives

Membrane PPase, combining in one polypeptide the energy-supplying and transporting modules, is among the simplest ion pumps found in nature. Recent studies have revealed many varieties of this transporter in plants, bacteria, archaea, and protists, allowing a thorough understanding of mPPase evolution. Vast literature on using mPPase to increase stress resistance of bacteria and agricultural plants is available. Furthermore, mPPase absence in humans makes it a promising drug target in pathogenic protists and bacteria. However, a full understanding of the coupling mechanism of mPPase still needs thorough structural and functional studies. These studies should include the determination of the physiologically relevant structures containing substrate and product in only one subunit of the dimer. The currently available structures contain them in both subunits, hindering understanding of energy transduction within the protein in the catalytic cycle. Determining the tertiary structure and transport stoichiometry of Na^+^,H^+^-PPase would shed light on the unusual relationship between Na^+^ and H^+^ transport in the concurrent transport of these cations under physiological conditions. Future biochemical characterizations should determine the number, identity, and role of the Na^+^-binding sites in Na^+^-transporting mPPases and Na^+^-regulated H^+^-transporting mPPase [67]. These studies should also explain the role of K^+^ in mPPase, in its Na^+^-transport function, in particular.

## Data Availability

Not applicable.
